# Questions Raised at the Private Visit

**Published:** 1914-03-07

**Authors:** 


### THE KING AND QUEEN AT ST. THOMAS'S HOSPITAL.

### Questions Raised at the Private Visit.

ONCE more the King and Queen have shown their great practical sympathy with the work of our voluntary hospitals by a long visit to St. Thomas's Hospital. Shortly before noon on Monday last a message was sent by the Equerry that at 3 P.M. the King and Queen would pay a strictly private visit to the hospital, and desired no one else but the Secretary and Matron to receive them.

### IN THE GOVERNORS' HALL.

A minute or two before the time announced the motor arrived at the main entrance, Lambeth Palace Road, their Majesties being accompanied by Vice-Admiral Sir Colin Keppel and Lady Eva Dugdale. The orders expressed by the Equerry were strictly adhered to, and only the Secretary, Mr. G. Q. Roberts, and the Matron, Miss Lloyd-Still, were present to receive their Majesties on the main steps of the building. As the Surgical Registrar, Mr. S. H. Rouquette, M.S.Camb., happened to be passing at the time he was invited to accompany their Majesties to explain any point that might be necessary in connection with any surgical work in the hospital with which he might be familiar. The King and Queen having expressed a desire first of all to see the Governors' Hall they were conducted thither, and were much interested by the coats of arms, including that of the Abbey of Bermondsey, St. Mary Overie, Peter de Roche (Bishop of Winchester), who re-endowed the hospital in 1213, Bishop Ridley, and Edward VI., who granted the Charter under which the hospital at the present time carries on its work, and who restored its fortunes after the sequestrations of the time of Henry VIII. The series of large coats of arms on the big memorial window is completed by those of Charles Gassiot, the most liberal benefactor of the hospital in modern times. The smaller windows hold the coats of arms of all the presidents of the hospital from the year 1553 to the time of the president *in esse*, the Duke of Connaught and Strathearn, K.G. In the Grand Committee-room the Queen was particularly interested in the scarves worn of old by the surgeons when on ceremonial duty in the hospital, and by the photographs or prints of past distinguished physicians and surgeons of the hospital, amongst whom were Mead, Cheselden, etc.

### THE KING ON ACCIDENTS AND LEAD POISONING.

The first ward visited was City Ward, which a liberal contribution of £10,000 from the Mercers' Company enabled the governors to open as a special ward for the reception of accidents and emergency cases which it is not considered desirable to admit direct to a clean surgical ward. Their Majesties spoke to various patients, and were very sympathetic with a little boy who had been run over by a motor-car, the King expressing regret at the fact that so often these accidents were caused by children running across the road so late that it was impossible for the drivers to avoid them, however careful they might be. The King particularly noted the advantage of two small wards attached to the entrance lobby of City Ward for the reception of delirious cases or others who, through drink or other cause, come in very noisy or very dirty, and likely to disturb other patients in the wards.

Their Majesties next went to the first floor, and in the Medical Ward the King made many inquiries of a painter there suffering from lead-poisoning. "Could nothing be done to avoid this by wearing gloves?" asked the King. "I am afraid, Sir," said the man, "the employer wouldn't appreciate a workman in kid gloves."

In Edward Ward the patients are under the charge of Mr. C. A. Ballance, F.R.C.S., M.V.O., surgeon to the Metropolitan Police. The King was naturally recognised, and a hearty round of cheers was given by the patients as he left the ward. Another surgical ward, Albert Ward, was visited, and both King and Queen stepped out on to the balcony, which is a feature of each ward of the hospital, and spoke to the patients lying there, two of whom, his Majesty was informed, slept there night and day—surgical tuberculosis cases.

The Louis Jenner Research Laboratory—director, Dr. L. S. Dudgeon, F.R.C.P.—was next visited, and the close relation of the work of the scientists of this laboratory with the work of the clinical physicians in the wards, the method of reference for investigation and inquiry into the nature of disease, was explained to their Majesties, who, in their tour of the laboratories, made very sympathetic inquiries from Dr. Gardner, the research scholar of the hospital, on the nature of the investigations he was carrying on at the present time. In Florence Ward, Dr. Hawkins, the senior physician, was taking a large class of students for the afternoon clinical demonstration, in which their Majesties showed warm interest. As the King noted one of the students was on crutches. It was explained that he, a Mexican, played wing three-quarter in the Rugby team, and had broken his leg in the cup tie match against Guy's, in which match St. Thomas's led for sixty minutes, but in the last twenty minutes, with two men injured, they were unable to prevent Guy's scoring freely. "I fear you will not play again this season," said the King. "No, Sir, but next season, I hope." Many patients in this ward were visited and were evidently greatly cheered by the sympathetic words of their Majesties. Both the children's wards, which are so well known for the picture tiles with which the walls are covered, the gift of various donors, such as Miss Lilian Holland, Miss Jewesbury, H.H. Ras Makonnen, and Mr. James Douglas, of Montreal, were visited, the Queen making very full inquiries from the sister of each of these wards into the personal circumstances of most of the children.

### THE KING AND THE PLENUM SYSTEM.

His Majesty particularly noted the fittings of the ward and the large glass and iron ambulance for dressings, which is in use there. The King was much interested in hearing the fact that an attempt had been made when this ward was constructed to use the plenum system of ventilation, but after a full and careful trial the plenum system had been abandoned, and natural ventilation depending on the bountiful supply of fresh air at St. Thomas's is in use in this ward. The clinical lecture theatre and the magnificent North operating theatres were visited, where the King noted the air, and asked if these rooms were on the plenum system, as indeed they are. An explanation was given to the effect that an order has already been given by the governors for the fitting of a new system of ventilation in these theatres with a view to securing a more bountiful supply of thoroughly sterilised air. The next point of interest which their Majesties had expressed a desire to see was the Maternity Ward, named after the Queen, and well known now both in the hospital and in the district where it is so much appreciated as "Mary Ward." To reach this conveniently from the theatre it was necessary to pass along the outside corridor on the second floor, from which a fine open view of the neighbourhood is obtained. Building operations are going on there in which their Majesties took great interest. A new operating theatre is being constructed for the sole use of the Obstetric Ward. Every detail in connection with the work of the Maternity Ward was inquired into and explained. Practically every patient in the ward was sympathetically talked to by the King, and all were proud to exhibit their contributions in the shape of a small hostage to fortune. When one poor mite was presented and described as twenty-four days old, "Twenty-four hours, Nurse, you must mean," said the King. "No, Sir, twenty-four days. This is one of twins prematurely born; the stronger twin has already been sent away to Dr. Barnardo's Home, where it is being well looked after." The King was much interested in the wide extent of the social work carried on through the Lady Almoner's Department in connection with every patient coming to St. Thomas's Hospital, not only as a preventive to the abuse of the charity, but to aid those poor sufferers in their hard struggles, especially during times of convalescence. The Preliminary Training School for Nurses was visited and the course of tuition explained, many of the rooms in the Nurses' Home being inspected.

### Visit to the Pay Wards.

Before leaving their Majesties went into St. Thomas's Home for Paying Patients, where they inspected the operating theatres and the arrangements in each of the small rooms there, and expressed their sympathy with many of the patients. Very warm appreciation of the hospital and its work and their pleasure at their visit was expressed. A very hearty round of cheers was given by the large number of students and nurses from the School of Massage and Swedish Remedial Exercises, who had assembled in the quadrangle between the hospital and the Nurses' Home, as the motor left the hospital grounds.

This first visit of the King to one of our most ancient hospitals will be highly appreciated by the governors. It was a purely informal and personal visit, but in the course of it their Majesties conveyed to all connected with the hospital with whom they came in touch the keen interest they both take in the hospital, and displayed a remarkable technical knowledge of the work in the course of a visit which the King said was "one paid to see the ordinary work being carried on."

